# Energy-Efficient Receiver-Driven Wireless Mesh Sensor Networks

**DOI:** 10.3390/s110100111

**Published:** 2010-12-23

**Authors:** Daichi Kominami, Masashi Sugano, Masayuki Murata, Takaaki Hatauchi

**Affiliations:** 1 Graduate School of Information Science and Technology, Osaka University, 1-5 Yamadaoka, Suita-shi, Osaka 565-0871, Japan; 2 School of Comprehensive Rehabilitation, Osaka Prefecture University, 3-7-30 Habikino, Habikino-shi, Osaka 583-8555, Japan; 3 Fuji Electric Systems, 1 Fujicho, Hino-shi, Tokyo 191-8502, Japan

**Keywords:** sensor network, intermittent operation, simulation, mesh network

## Abstract

A major challenge in wireless sensor networks research is energy efficiency. In the intermittent receiver-driven data transmission (IRDT) protocol, which aims at saving energy, communication between two nodes commences when multiple receiver nodes transmit their own IDs and the sender nodes receive them. This protocol can be used to construct a mesh network which is robust against node failure and wireless channel fluctuations. In our work, we improve this protocol by implementing a collision avoidance method for control packets. First, we refer to the probability of control packet collision as a function of the intermittent interval. We then introduce procedures to determine the interval which decreases or minimizes this probability. Afterwards, we include a data aggregation mechanism into IRDT to reduce data transmission frequency and the occurrence of control packet collisions. Through computer simulation, we show that IRDT can offer greater reduction of the average energy consumption compared with RI-MAC and X-MAC, especially at small loads, and we also demonstrate that IRDT with collision avoidance for control packets can attain higher performance than the original IRDT. This method ensures a packet collection ratio of more than 99% and an average energy consumption 38% lower than that of EA-ALPL and 90% lower than that of the original IRDT.

## 1. Introduction

Recently, due to advances in wireless and micro-electromechanical (MEMS) technologies, extremely small sensor nodes featuring wireless communication facilities have been developed, and as a result wireless sensor networks have received considerable attention. Sensor networks are particularly useful for a wide range of applications as they possess sensing capabilities without the need for implementing a centralized infrastructure.

However, some critical technical problems still need to be resolved in wireless sensor networks, one of which is the energy efficiency of sensor nodes with limited battery life. There are various approaches to the improvement of energy efficiency, for example, miniaturization of the sensor nodes, media access control (MAC) with sleep control, and multi-hop routing [1–4]. In this paper, we use one of the MAC layer approaches, namely, intermittent operation. In particular, considerable amounts of energy can be saved through intermittent operation, in which wireless nodes sleep to save power and wake up periodically to transmit or receive packets. Here, we refer to this wake-up interval as ‘intermittent interval’. This power-saving operation is based on the fact that sleeping nodes consume considerably less energy than idling nodes [5]. In intermittent operation, nodes must control wake-up times in order to communicate with each other.

Control methods for intermittent operation are classified into two types: synchronous [3,6,7] and asynchronous [4,8–10]. A synchronous method uses a beacon to maintain synchronization between intermittent operations. The advantage of synchronization is that the delay between the waking up and data transmission states is shorter for sender nodes, which reduces energy consumption. The disadvantage is that regular beacon transmission consumes large amounts of energy and causes interference. Furthermore, all nodes must use a fixed intermittent interval. In the asynchronous method, each node can communicate with other nodes at any given point in time. The advantage of this method is that there is no traffic overhead for synchronization, which reduces energy consumption and results in a highly scalable network. However, in the asynchronous method, the sender node waits in an idle listening state until the receiver node awakens, which increases the consumption of energy in sender nodes. In order to save energy, each node must use long intermittent intervals to reduce its own duty cycle; however, this also results in the consumption of large amounts of energy in sender nodes. In terms of the overhead for control of synchronization with other nodes, the latter is superior in terms of saving energy and enhancing scalability in systems with low packet generation rates. Here, we classify the asynchronous control method into two subtypes, namely, sender-driven and receiver-driven type, depending on whether the sender or the receiver initiates communication. In either type, packet collisions must be controlled since nodes can commence communication at any given point in time in an asynchronous control method.

We focus on the smart meter system [11] as an application that requires a high packet collection ratio and operates for several years without the need for replacing the battery in a situation where the data generation frequency is comparatively small. Hence, in a case where sensor nodes with intermittent operation form an ad hoc network, the asynchronous method is best suited for our purposes. In addition, our target application must be adaptable to occasional burst traffic conditions in order for a network employing this system to be usable in various security management systems.

**Figure 1.**
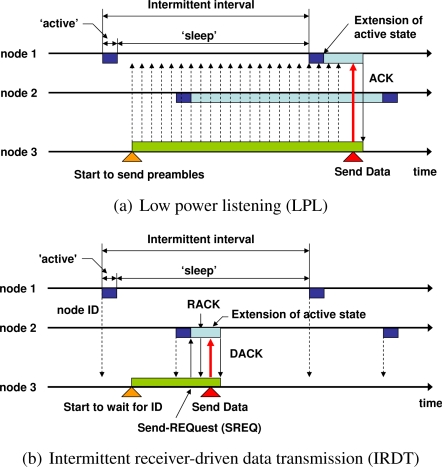
Asynchronous intermittent transmission methods.

The *low power listening* (LPL) protocol is a sender-driven asynchronous type of ad hoc network system [9]. The basic intermittent operation of LPL (B-MAC [4]) is shown in Figure 1(a). Receiver nodes 1 and 2 intermittently check the state of the channel. If the channel is idle, they return to the ‘sleep’ state, and if it is busy, they enter the ‘data wait’ state. After receiving data packets intended for them, nodes return ‘acknowledge’ packets. For instance, when node 3 is ready to send data to node 1, it continuously sends preamble packets for a period of time which is longer than the intermittent interval in order to activate the channel. After sending preamble packets, node 3 sends a data packet. However, there are a number of restrictions in this protocol; for example, when the intermittent interval is comparatively long, each sender node occupies the channel for a long period of time while transmitting preamble packets. This occupation of the channel interferes with the communication between neighboring nodes. As another example, each sender node transmits data packets only toward a specific node, which entails poor tolerance with regard to node failure.

In order to overcome these drawbacks of LPL, we proposed the *intermittent receiver-driven data transmission* (IRDT) protocol in our previous work [12]. IRDT is one of the receiver-driven MAC protocols where communication between two nodes commences when receiver nodes transmit their own IDs and sender nodes receive them. IRDT addresses some of the restrictions of LPL; for example, it does not occupy the channel when the intermittent interval is long, and it can select a neighboring node as a receiver node from multiple neighbors, which can be used for constructing a mesh network at the MAC layer. In IRDT, receiver nodes transmit small packets containing their own ID (denoted as ID packet) periodically and intermittently. Sender nodes wait for an ID packet of a receiver node and when it acquires such an ID packet from an appropriate receiver, it transmits a send request (SREQ) packet to establish a link. Note that we have developed IRDT as a protocol which has actually been implemented in meter products [12]. Furthermore, we are currently proposing this technique to IEEE 802.15 Task Group 4 as part of a standard protocol for smart meter systems [11].

In this paper, we clarify the performance characteristics of IRDT by comparing them with those of X-MAC protocols and another receiver-driven MAC protocol (RI-MAC) through computer simulations. The long-term operation of IRDT is examined by comparing the energy consumption under conditions of low data incidence. Additionally, we implement improvements in IRDT by decreasing the incidence of control packet collisions. Control packet collisions are classified into two types, namely ID collisions, occurring between ID packets and other packets, and SREQ collisions, occurring between multiple SREQ packets. Such collisions drastically reduce the performance of IRDT, and we discuss them in detail in Section 4. Furthermore, we propose a simple and effective routing algorithm for mesh networks with IRDT, as well as novel improvement mechanisms for IRDT, and evaluate the impact of these improvements.

The rest of this paper is organized as follows. In the following section, we present some related work, and in Section 3, we describe the details of IRDT. In Section 4, we discuss control packet collisions in IRDT and some approaches to avoiding it. Finally, we present the simulation results in Section 5 and our conclusions in Section 6.

## 2. Related work

In this section, we present some MAC protocols for intermittent asynchronous transmission and demonstrate the essential differences between sender-driven MAC and receiver-driven MAC.

There are various approaches to media access control for intermittent asynchronous transmission. **B-MAC** [4] is the basis of LPL protocols as presented in Figure 1(a). In LPL, receiver nodes intermittently probe the state of the channel. As mentioned above, there are various problems associated with this LPL protocol; for instance, when the intermittent interval is comparatively long, each sender node occupies the channel by transmitting preamble packets for a period of time which is longer than the interval, thus interfering with any transmission from neighboring nodes. Moreover, the preamble packets transmitted from the sender consume the energy of unrelated receivers, which is known as “overhearing problem”. Another problem is that each sender node has only one specific receiver.

Energy-aware adaptive low power listening (**EA-ALPL** [9]) is based on B-MAC. The procedure followed by receivers and senders in EA-ALPL is the same as the one shown in Figure 1(a), however, each node reconfigures its intermittent interval and adapts it to changes in traffic in order to attain higher energy efficiency. For high energy efficiency, the next hop selected by a sender node is the receiver which has the minimum hop count from the sink node. When there are multiple receiver candidates with minimum hop count, a sender node selects one of the most preferable nodes in accordance with the cost function of the intermittent interval and the sensing activity of neighboring nodes. The sensing activity is a Boolean variable, and it is determined by the sensing frequency of a node. In order to select a receiver, nodes regularly exchange information regarding the sensing activity and their own intermittent interval.

**Figure 2.**
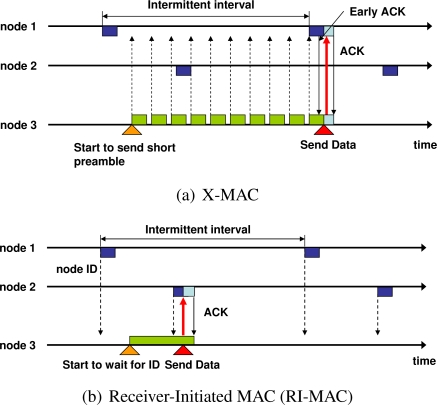
Other intermittent transmission methods.

**X-MAC** [8] was designed to solve the overhearing problem of B-MAC. In order to prevent the preamble packets of the sender in B-MAC from occupying the channel, X-MAC continuously transmits short preamble packets to which the ID of the receiver is appended. The operation of X-MAC is shown in Figure 2(a). The receiver node replies with an early acknowledge (early ACK) packet when the ID added to the short preamble corresponds to its own ID. The sender node transmits a data packet after receiving this early ACK and waits for the acknowledge packet for the data. Receivers that detect unrelated short preambles can resume their state of sleep soon after the end of the reception. Thus, the overhearing problem generated by continuous transmission of preambles during intermittent intervals in B-MAC can be solved.

Although various receiver-driven asynchronous MAC protocols have also been proposed, most of them either assume that all nodes are active and can receive packets at any time, or that they use multi-channel access for transmitting packets [10,13,14]. In [13], receiver-driven media access control with a single channel, named “receiver initiated multiple access” (**RIMA**), is proposed. RIMA employs a collision avoidance handshake mechanism with CSMA/CA and obtains a reasonable throughput; however, this protocol does not use intermittent operation since it does not consider energy consumption.

In [10], two generic intermittent asynchronous MAC protocols are proposed, namely, Transmitter Initiated CyclEd Receiver (**TICER**) and Receiver Initiated CyclEd Receiver (**RICER**). The procedure of sending and receiving data in RICER is similar to that in IRDT, where receiver nodes periodically transmit ID packets. However, unlike the procedure in IRDT described in Section 3, a sender node in RICER transmits a data packet after obtaining an ID packet. Furthermore, two channels are used for communication, and a sender uses only one receiver in RICER. In contrast, IRDT uses a single channel, which simplifies the implementation and ensures a highly reliable system. However, single-channel access causes control packet collision.

Receiver-initiated MAC (**RI-MAC**) is also a receiver-driven MAC protocol, and thus it is similar to RICER [14]. In RI-MAC, a sender also transmits a data packet after receiving an ID packet, however, RI-MAC uses a single channel for the transmission of packets (Figure 2(b)). In order to avoid packet collisions, RI-MAC only uses collision detection and exponential backoff. Also, in terms of the routing algorithm, the authors of this protocol used minimum hop routing. IRDT uses an adaptive intermittent interval, whereas both RICER and RI-MAC use a fixed value for the intermittent interval. Such an adaptive interval can avoid packet collisions and can attain higher performance. In this paper, we propose a simple and effective routing algorithm for IRDT which is considered for mesh networks in an effective and efficient manner.

Here, an essential difference between IRDT and LPL is that nodes in intermittent operation mode transmit packets or listen to the channel, which can also be considered an essential difference between the sender-driven method and the receiver-driven method. In our previous research, we demonstrated the impact of this difference on the performance.

## 3. Intermittent Receiver-Driven Data Transmission

### 3.1. MAC Protocol

In IRDT, each receiver sends its own ID to inform other nodes that they are ready to receive a data packet. A sender node waits for a receiver ID, and when it acquires an ID from an appropriate receiver, it establishes a link with it by returning an SREQ packet. After obtaining an acknowledge packet for SREQ (RACK), the sender transmits a data packet and terminates the communication upon receipt of an acknowledge packet for the data (DACK). Carrier sense multiple access with collision avoidance (CSMA/CA) is used for sending packets. However, especially when a node transmits an ID packet or an SREQ packet, it terminates the transmission of those packets if the channel condition is busy. If the channel is idle, it transmits an ID packet or an SREQ packet after a random backoff period. Otherwise, when it transmits a data packet, a RACK packet, or a DACK packet, a binary exponential backoff mechanism is utilized.

**Figure 3.**
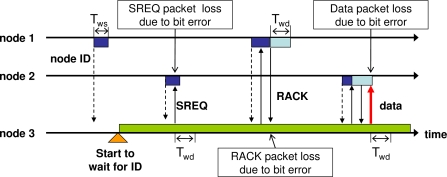
*T_ws_* and *T_wd_* timers in IRDT.

Here, all nodes contain two timers, which are set immediately before starting to wait for an SREQ packet, a RACK packet, a data packet, or a DACK packet. *T_ws_* is the time allocated for waiting for an SREQ packet following the transmission of an ID packet. Furthermore, *T_wd_* is the time allocated for waiting for a data packet, a RACK packet, and a DACK packet. After the transmission of a RACK packet, an SREQ packet, or a data packet, respectively, as shown in Figure 3. If a time *T_ws_* passes before receiving an SREQ packet after the transmission of an ID packet, the receiver node enters sleep mode, as shown in the figure. The receiver node also enters sleep mode if the period *T_wd_* before receiving a data packet after transmitting a RACK packet extends beyond a certain limit. On the side of sender nodes, if a RACK packet and a DACK packet are not received from the receiver after a lapse of *T_wd_*, they begin to wait for reception of another appropriate ID packet. Note that, for the CSMA/CA backoff algorithm, *T_ws_* is shorter than *T_wd_*.

The decision of the sender regarding whether to send an SREQ packet is taken on the basis of its routing protocol. In this way, a sender node can select a receiver node flexibly, which can enhance the communication reliability and save considerable amounts of energy. Therefore, in the network layer, the routing protocol should be designed to use multiple receiver nodes in a flexible and efficient manner. A specific example is shown in Figure 1(b), where receiver nodes 1 and 2 are in intermittent operation mode. Sender node 3 checks the ID received from node 2 and accepts node 2 as an appropriate receiver.

### 3.2. Routing Protocol

The routing protocol of IRDT is based on the distance vector routing protocol. All nodes have routing tables and a routing function for deciding on the transmission of an SREQ packet.

A routing table contains hop counts from the node which has created the table to all nodes in the network. In order to create its own routing table, each node must exchange its table with its neighbors. In IRDT, all nodes periodically wake up and wait for ID packets for a short period of time, which, however, is longer than the intermittent interval. When a node receives an ID packet within this period, it registers on its routing table that the hop count to the sender of the ID is one. We refer to this interval as ‘sampling interval’ (denoted by *T_si_*).

The routing algorithm for IRDT must be based on multi-hop routing, and therefore each node conducts the relay processing of the packet. Although minimum hop routing is preferable for the purpose of minimizing energy consumption, in some situations nodes cannot utilize the optimal routing due to poor radio wave conditions or failure of certain nodes. Therefore, for higher flexibility, the routing algorithm considers alternatives to the minimum hop route. Here, we define forward nodes, sideward nodes, and backward nodes. A node whose hop count from the sink node is *H* classifies its adjacent nodes as shown below.

**Forward nodes:** Adjacent nodes whose hop count from the sink node is *H* − 1.
**Sideward nodes:** Adjacent nodes whose hop count from the sink node is *H*.
**Backward nodes:** Adjacent nodes whose hop count from the sink node is *H* + 1.

**Figure 4.**
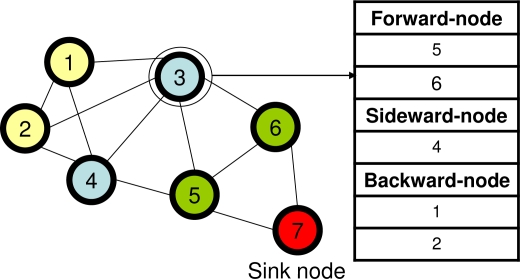
Classification of neighboring nodes at node 3.

Figure 4 shows an example of this classification of neighboring nodes.

**Figure 5.**
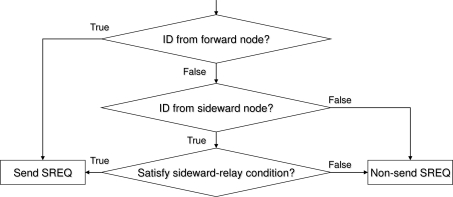
An example of a routing function.

The routing function is a logic function that utilizes a routing table. Sender nodes decide whether to return an SREQ packet in accordance to this function, an example of which is shown in Figure 5. The function in Figure 5 assumes the minimum hop routing; however, detours are also used when the condition of sideward relay is satisfied.

Here, we define communication failure as a situation in which the sender cannot obtain a RACK and a DACK from the receiver. For minimum hop routing with detours, the sender node prefers forward nodes as receivers, and sideward nodes are selected if communication with all forward nodes fails. In order to prevent routing loops, all data packets have a time to live (TTL) field. The TTL is decremented by one only when a receiver node has received a data packet from a sender node. When the TTL becomes zero and the receiver is not the destination of the data packet, the data packet is discarded. No sender node selects a sideward node or a backward node if this results in loss of data packets due to the TTL mechanism.

## 4. Control Packet Collision

**Figure 6.**
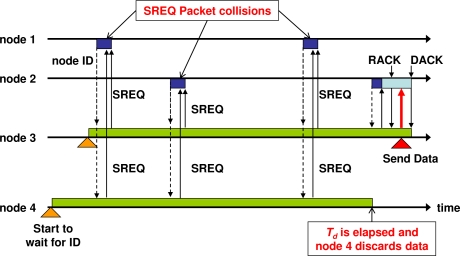
Recurring SREQ collisions; A major cause of excessive energy consumption in sender nodes.

In this section, we discuss the control packet collision problem in IRDT together with some novel approaches to resolving it. One problem related to IRDT is collisions between ID packets and other packets, as well as collisions between SREQ packets, which we refer to as ‘ID collisions’ and ‘SREQ collisions’, respectively. All nodes send ID packets periodically, and therefore ID packets can collide with other packets. Regarding SREQ collisions, the sender node returns an SREQ packet when an ID packet from a forward node arrives, as described in Section 3.2. Thus, if more than one sender receives an ID from a forward node, the sender nodes return SREQ packets simultaneously, the packets collide with each other. In this case, the sender nodes remain awake in wait for another ID, and as a result their energy consumption increases. Furthermore, SREQ collisions are in danger of recurring at nodes that are the only forward nodes for their backward nodes. For example, this ‘recurring SREQ collision’ often occurs at the sink node, which is the only forward node for its neighbor nodes. After an SREQ collision occurs at the sink node, more than one neighbor node still contains data packets. This causes another SREQ collision following the ID transmission by the sink node. Due to the scheduled timer for discarding data (set to *T_d_*) built into all nodes, recurring SREQ collisions eventually cease. Since the sender continues waiting for an ID packet until the sender receives a DACK packet from a receiver, recurring SREQ collisions lead to large energy consumption, as shown in Figure 6. For the above reasons, a reduction of the respective rates of ID and SREQ collisions (collectively denoted as “control packet collisions”) is meaningful in terms of energy efficiency.

Next, we describe the influence of the intermittent interval on the probability of packet collisions, as well as the procedure for determining a proper intermittent interval which decreases this probability. Changing the intermittent interval affects the following two aspects:

1. Probability of SREQ collisions
  This is the probability with which multiple nodes return SREQ packets simultaneously immediately after a receiver node sends an ID packet. Since SREQ collisions are caused by data packet congestion, a longer intermittent interval increases this probability. If SREQ collisions occur, the energy consumption of the sender nodes increases due to retransmissions. Furthermore, such SREQ collisions can occur repeatedly.
2. Probability of ID collisions
  This probability corresponds to the likelihood that ID packets sent periodically by all neighboring nodes collide with SREQ or data packets. It is clear that a shorter intermittent interval increases this probability. As in the case of SREQ collisions, retransmissions increase energy consumption.

We propose three methods for resolving the control packet collision problem, namely, reactive and proactive control of the intermittent interval and data aggregation. A protocol using the reactive method starts avoiding SREQ collisions soon after the first SREQ collision occurs. The advantage of this method is adaptability to changes in the network topology and the packet generation rate. In comparison, in the proactive method, the optimal intermittent interval which minimizes the sum of the respective probabilities for SREQ collisions and ID collisions is obtained analytically, where each node knows its own traffic load. We refer to this intermittent interval as the “proper interval” (denoted as T*). Finally, data aggregation can be used to decrease the number of data packet transmissions for each node, which can decrease the probability of SREQ collisions.

### 4.1. Collision Avoidance with the Reactive Interval Setting

**Figure 7.**
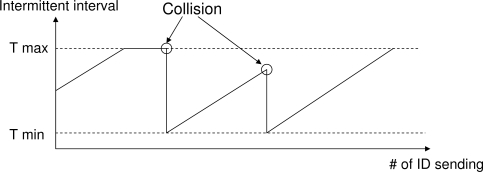
Dynamic control of the intermittent interval as proposed in [15].

SREQ collisions are caused by two factors, one of which is the disagreement between the transmission capacity and the load of a node. The maximum number of packets that a node can receive per unit time corresponds to the number of IDs the node sends per unit time. Therefore, as the intermittent interval of a node is shortened, the amount of data that a node can receive increases. When the load exceeds the processing performance of the node, multiple SREQ packets are sent, and collision occurs. Accordingly, in the reactive method, each node sets its ID transmission interval dynamically. Nodes determine that their loads are high when collisions are detected while they are waiting for an SREQ packet. In this case, they set their own intermittent intervals to *T_min_*. If an SREQ collision is not detected, the nodes gradually increase their intermittent interval to *T_max_* at increments of *T_i_* after every transmission of an ID in order to reduce the duty cycle (Figure 7). Regarding *T_max_* and *T_min_*, although a longer *T_max_* decreases the duty cycle of the node, it affects its neighbors by increasing the interval of waiting for an ID. In contrast, while a shorter *T_min_* improves the transfer performance, it interferes with communication between other nodes.

The other factor is the priority of forward nodes as receivers. As described in Section 3.2, when a sender node receives an ID packet from its forward node, it transmits an SREQ packet. Therefore, when more than one hidden node is ready to send data to the same receiver, whenever the receiver transmits an ID, an SREQ collision occurs. In addition, even if there are no hidden nodes, SREQ packets will collide if they are transmitted simultaneously. At nodes which are the only forward nodes for a large number of sender nodes, such as the sink node, SREQ collisions occur repeatedly, as mentioned before in the section regarding recurring SREQ collisions. In order to solve this problem, it is necessary for sender nodes to ignore the ID packet of their forward nodes in a random fashion. Therefore, if a node fails to transmit a packet to all its forward nodes, which is a situation described as ‘communication failure’ in Section 3.2, it ignores IDs from the forward nodes with a fixed probability denoted by *P_f_*.

As *P_f_* becomes larger, sender nodes tend to transmit data packets to sideward nodes. Thus, a large *P_f_* leads to an increase in both the number of data relays and the period of waiting for ID packets from sender nodes. We utilize the concept of disregarding ID packets with a certain probability for selecting the appropriate *P_f_*. Although this additional process cannot prevent initial collisions, once a collision occurs, each sender node autonomously avoids further collisions.

### 4.2. Collision Avoidance with Proactive Interval Setting

#### Analytical derivation of the probability of control packet collision

In analyzing the probability of control packet collision, we introduce the following assumptions.

**Figure 8.**
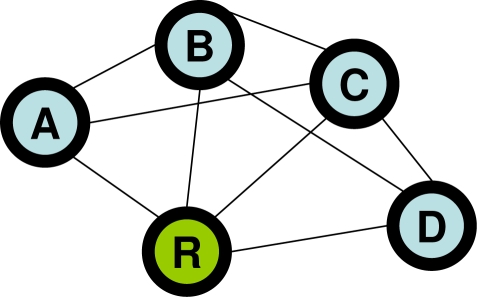
Simple network model.

All nodes possess complete information about the network topology and contain a static routing table based on this information. Here, we use the topology shown in Figure 8, where node *R* is a sink node. Thus, the forward node of node *A* is node *R*, and its sideward nodes are node *B* and node *C*.
Each sensor node generates a data packet in accordance to a Poisson process with intensity λ, and subsequently sends the data to the sink node. In addition, when nodes forward data, they always select forward nodes, and any forward node is equally likely to be chosen as the receiver.
When packet collisions occur, the receiver of the packets always discards all packets involved in the collision.
Each node sends ID packets at a regular intermittent interval denoted as *T*. Moreover, all nodes perform the “clear channel assessment” (CCA) procedure when sending any type of packet. Neither ID packets nor SREQ packets are transmitted if the CCA has indicated that the wireless channel is busy. If the wireless channel is idle, nodes transmit an ID or an SREQ packet after a random backoff period of time. After it is ensured that receivers can obtain SREQ packets correctly, collisions between data, RACK and DACK packets and other packets occur less frequently. However, if a collision occurs, a receiver must wait for the following ID packet, which increases the total amount of time spent by the affected sender node in waiting for an ID packet. Therefore, data packets, RACK packets and DACK packets are transmitted by using binary exponential backoff in order to prevent collisions with other packets (especially ID packets).

From the above assumptions, we can calculate *G*(*R*), which is the approximate average number of data packets received by node *R* in one second. *G*(*R*) depends on the number of backward nodes for node *R* and its traffic load. Here, we define *N_b_*(*R*) as the set of backward nodes of node *R* and |*N_f_* (*n*)| as the number of forward nodes of node *n*. The probability with which a node (denoted as *n*) selects node *R* as its receiver is $\frac{1}{\left|N_{f}\left(n\right)\right|}$, and therefore *G*(*R*) is expressed as follows:

(1) $$G\left(R\right)=\sum_{n\in N_{b}\left(R\right)}\frac{1}{\left|N_{f}\left(n\right)\right|}\left\{G\left(n\right)+\lambda \right\}.$$

SREQ collisions occur when two or more neighboring nodes send SREQ packets simultaneously. We assume that all nodes use the CSMA/CA mechanism, which can reduce the number of SREQ collisions.

However, SREQ collisions can still occur, unless there are no hidden nodes, since SREQ packets can be returned at once. In the CSMA/CA mechanism with exponential backoff, the number of time slots chosen at random by each node is 2*^BE^*, where *BE* is a moderate integer value. If the wireless channel is idle, the sender node transmits an SREQ packet (or an ID packet) after a CCA and a random backoff period, as described in Section 3.1. In this regard, a time slot with a range of 2*^BE^* is utilized for the random backoff period. Here, we assume that node *R* receives the same number of data packets from each of its backward nodes. Therefore, the probability with which a node returns an SREQ packet upon receiving an appropriate ID can be expressed as 1 − *e^−G_b_(R)T^*, where *G_b_*(*R*) is $\frac{G\left(R\right)}{\left|N_{b}\left(R\right)\right|}$. Furthermore, the probability with which the node does not return an SREQ packet can also be expressed as *e^−G_b_(R)T^*. *P_SREQ_*, which is the probability with which SREQ collisions occur, is also the probability with which at least two neighboring nodes of node *R* receive a data packet. However, the CSMA/CA mechanism cannot avoid SREQ collisions when node *R* sends an ID packet. Thus, *P_SREQ_* can be calculated as follows:

(2) $$P_{\mathrm{SREQ}}=1-\sum_{k=0}^{\left|N_{b}\left(R\right)\right|}C\left(R,k\right)e^{-\left(|N_{b}\left(R\right)|-k\right)G_{b}\left(R\right)T}{\left(1-e^{-G_{b}\left(R\right)T}\right)}^{k},$$

where *C*(*R*, *k*) indicates the number of combinations of *k* different nodes out of *N_b_*(*R*), which addresses the hidden node problem under CSMA/CA.

Here, we consider only the case where *k* is less than three because the term *e*^−(|*N_b_*(*R*)|−*k*)*G_b_*(*R*)*T*^ (1 − *e*^−*G_b_*(*R*)*T*^)*^k^* is exceedingly small and can be ignored for large *k*. *C*(*R*, *k*) is defined as follows:

(3) $$C\left(R,k\right)=\{\begin{array}{ll} 1 & \left(k=0\right)\\ \left|N_{b}\left(R\right)\right| & \left(k=1\right)\\ \frac{2^{\mathrm{BE}}-1}{2^{\mathrm{BE}}}h\left(R\right) & (k=2), \end{array}$$

where *h*(*R*) is the number of couples of nodes out of *N_b_*(*R*) in relation to the number of hidden nodes.

Next, we target collisions of ID packets at node *R*. A collision of ID packets occurs when ID packets are sent by the neighbors of node *R* while node *R* is receiving an SREQ or a data packet. Note that it is not necessary to consider the backoff time slot of CSMA/CA as discussed in *P_SREQ_* since ID packets are rarely transmitted simultaneously by multiple nodes. Here, we define *H*(*R*) as the average number of hidden nodes for node *R* for the time when node *R* is receiving SREQ or data packets. *H*(*R*) is represented as follows:

(4) $$H\left(R\right)=\frac{1}{\left|N_{a}\left(R\right)\right|}\sum_{n\in N_{a}\left(R\right)}h\left(R,n\right),$$

where *N_a_*(*R*) is the set of adjacent nodes for node *R*, |*N_a_*(*R*)| is the number of elements of *N_a_*(*R*) and *h*(*R*, *n*) is the number of hidden nodes for node *n* included in *N_a_*(*R*).

The average interval for receiving ID packets while node *R* is receiving SREQ or data packets can be computed as $\frac{T}{H\left(R\right)}$ because *H* (*R*) nodes can send ID packets even while node *R* is receiving other packets. Here, we define *T_r_* as the reception time for SREQ and data packets, in which case the probability of ID collisions, denoted as *P_ID_*, is expressed as follows:

(5) $$P_{\mathrm{ID}}=\frac{T_{r}H\left(R\right)}{T}.$$

#### Procedure for determining the proper transmission interval

In order to determine the proper transmission interval, we modify Equation (2). Equation (2) shows the probability with which an SREQ collision occurs when an ID packet is sent by node *R*, and Equation (5) shows the probability with which an ID collision occurs when node *R* receives an SREQ or a data packet. Therefore, we introduce *P′_SREQ_* (the product of *P_SREQ_* and (*G*(*R*)*T*)^−1^), which corresponds to the probability with which an SREQ collision occurs when receiving an SREQ or a data packet (Equation (6)).

(6) $$P_{\mathrm{SREQ}}^{\prime }=\frac{1-\sum_{k=0}^{2}C\left(R,k\right)e^{-\left(2-k\right)G_{b}\left(R\right)T}{\left(1-e^{-G_{b}\left(R\right)T}\right)}^{k}}{G\left(R\right)T}.$$

Then, we can obtain T* by minimizing *P_CTRL_*, which is the probability of control packet collisions, as follows:

(7) $$P_{\mathrm{CTRL}}=P_{\mathrm{SREQ}}^{\prime }+P_{\mathrm{ID}}.$$

Unfortunately, an explicit expression of T* which minimizes Equation (7) cannot be given; instead, we can compute the approximate value of T* by calculating the minimum value of the sum and subsequently computing T* every 10 ms in the semi-open interval (0.0 s, 2.0 s].

**Figure 9.**
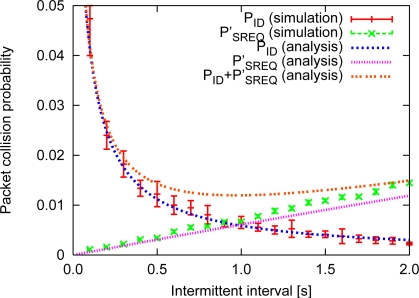
Probability of control packet collisions.

Figure 9 shows the results of the analysis and simulation of control packet collisions for the network topology shown in Figure 8, where λ = 0.024, *BE* = 3 and the error bar corresponds to the 95% confidence interval. From the results shown in Figure 9, it can be concluded that the analysis and the simulation of both *P_ID_* and *P_SREQ_* correspond rather well, which indicates that our analysis is correct. However, for *P_SREQ_*, as the intermittent interval becomes longer, the simulation results indicated superior performance than the analytical results due to the assumption that CSMA/CA can always prevent packet collisions, except in the presence of hidden nodes. In fact, CSMA/CA cannot completely avoid packet collisions even when two nodes are hidden with respect to each other. Also, SREQ collisions tend to occur more often as more backward nodes contain data packets. Therefore, when the packet generation rate is high, SREQ collisions occur more frequently. In an actual multi-hop network, a node sends data packets not only to forward nodes, but also to sideward nodes and backward nodes since *P_SREQ_* in an actual network is difficult to estimate. Moreover, the actual average number of data packets received in one second increases due to retransmissions.

### 4.3. Collision Avoidance with Data Aggregation

Data aggregation can reduce the number of data packet transmissions for each node. We assume that when a node aggregates *m* data packets, the size of the data packet increases *m* times, and the number *m* is appended to the ID packets in order to inform the receiver nodes about the identity of the sender node. Therefore, a larger *m* effectively decreases *G*(*R*) in Equation (6), and *P_SREQ_* also decreases. Unfortunately, it increases *T_r_* in Equation (5) as well as *P_ID_*. We present this trade-off in the following section.

Here, we demonstrate the strong effect of data aggregation with sideward nodes. In [15], we presented a method in which each node gives priority to forward nodes as appropriate receivers. This is caused by data transmissions toward sideward nodes, which increases both the number of data relays and the consumption of energy. When using data aggregation, however, relay with sideward nodes is more effective since data aggregation with both sideward and forward nodes greatly decreases *G*(*R*). SREQ collisions can occur between two or more nodes even if they are not hidden. This occurs when the random numbers for two nodes selected through the binary exponential backoff mechanism coincide. For example, if node 3 and node 4 in Figure 6 are not hidden nodes, an SREQ collision might occur. However, if data aggregation at these nodes is performed well, only one node contains the aggregated data packet, and no SREQ collision occurs. Moreover, data aggregation can resolve recurring SREQ collisions which occur when there is only one forward node, such as a sink node. In our previous research, we demonstrated that these repeated SREQ collisions cause an increase in energy consumption. If IRDT does not use data aggregation, repeated SREQ collisions continue to occur until the sending time expires. Specifically, when data aggregation is possible, the priority of the forward nodes is extended to sideward nodes which contain data packets. Whether sideward nodes receive data packets can be determined by adding this information to the ID packets.

We limit the size of the aggregated data packets for the reasons noted above, namely, a large value of *m* increases both *P_ID_* and the channel occupation time. We insert the number *m* into the ID packets in order to inform the receiver nodes about it, which can also be used to provide information about whether sideward nodes receive data packets. The use of this information prevents the data packet size from exceeding *m* times the original data size as a result of aggregation.

**Figure 10.**
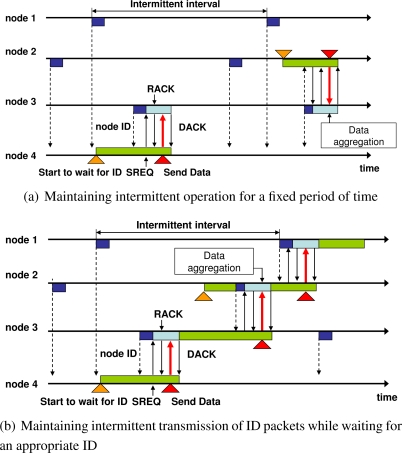
Data aggregation procedures in IRDT.

Here, two methods can be used to add the functionality of data aggregation to IRDT:

1. Maintaining intermittent operation for a fixed period of time: Sender nodes immediately begin to wait for an ID packet in IRDT when they receive or generate a data packet. At that time, data aggregation can be achieved by continuing their intermittent transmission of ID packets in order to receive data packets until the end of the fixed period of time without waiting for an ID packet, as shown in Figure 10(a). The node begins to wait for an ID packet when the size of the aggregated data packet reaches a certain predetermined size or a certain period of time passes.
2. Maintaining intermittent transmission of ID packets while waiting for an appropriate ID: In the current implementation of IRDT, the node which contains a data packet does not send an ID packet, although it is waiting for ID packets from other nodes. In order for sender nodes to receive data packets while waiting for an ID packet, they alternate the processes of transmitting ID packets and waiting for an appropriate ID packet, as shown in Figure 10(b). When they receive an SREQ packet, they perform data aggregation, and when they receive an ID packet from an appropriate receiver, they cease the aggregation and transmit an SREQ packet.

The first method decreases the data transmission frequency through aggressive data aggregation, while the second method aggregates data without increasing the delay time. In this paper, we focus on the first method in order to achieve higher energy efficiency.

## 5. Simulation Results

**Figure 11.**
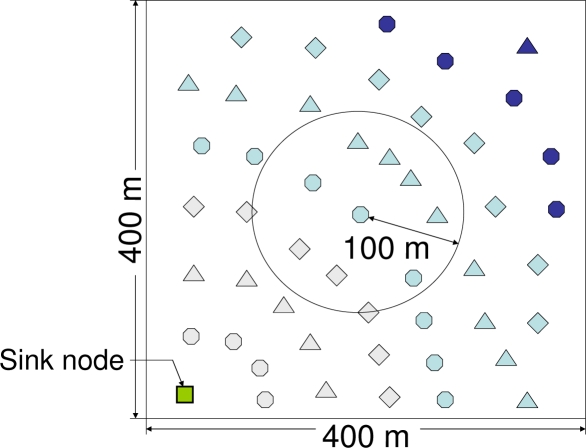
Network model.

In this section, we evaluate and compare the performance of IRDT, RI-MAC and X-MAC by using computer simulation. Also, we clarify the impact of collision avoidance for control packets. We devised a large-scale sensor network system composed of a large number of nodes as an application of the proposed method to our further studies. However, the ns-2 simulator, which is the most general simulation tool, does not scale well for such sensor networks, as discussed in [16]. Therefore, we prepared an event-driven simulation program written in Visual C++ for this experiment. Evaluation by using a general simulator that scales well for sensor networks is under consideration. Here, we use the network model shown in Figure 11, in which one sink node and 49 sensor nodes are deployed over 400x400 m^2^. In this figure, the sink node is represented as a square, and other shapes denote sensor nodes. The communication range of each node is 100 m, and the sensor nodes shown in the figure with the same shape and color have the same number of hops from the sink node. When modeling the network, we used the following assumptions:

- Static network topology
- A disk model is used in order to abstract away from any fluctuations in wireless communication
- The capture effect is not considered

In order to examine the impact of collision avoidance for control packets, we assume that the network topology is static. Regarding the model of communication between nodes, we employ the disk model, where the strength of the radio signals does not deteriorate, and unless packet collisions occur, a transmitted packet is assumed to be received for certain by the nodes within the communication range. In addition, our evaluation is performed on with conservative settings for the packet collision model in which both packets are always discarded if a packet collision occurs while a packet is being received.

Note that when another wireless communication model is utilized, the value of T* is varied with time, and therefore nodes should frequently exchange information about the network topology for the purpose of calculating *G*(*R*). Also, regarding the capture effect, even though the value of *P_SREQ_* appears to decrease slightly, SREQ collisions are of intrinsic importance in IRDT.

In our simulations, sensor nodes other than the sink node in the network generate data packets according to a Poisson process. Each sensor node transmits data to the sink node through a multi-hop relay, where the routing algorithm for IRDT in the simulation is described in Section 3.2. Here, data is collected after completion of the exchange of routing tables. Each node conducts CSMA/CA in order to avoid collisions with other packets. Before a node transmits an ID packet or an SREQ packet, it performs a clear channel assessment (CCA). If the channel is busy, it does not transmit a packet. In the case of other types of packet transmission, a node performs up to five attempts for binary exponential backoff of CSMA/CA. The initial size of the contention window is set to *W_min_* and incremented up to *W_max_*. All nodes use a data discard timer for preventing repeated SREQ collisions from occurring, where the timer is set to *T_d_*. The parameters are set as shown in Table 1. In particular, the *TTL* is set to *H* + 3 (*H* is the number of hops from the sink node) since extra relays increase the energy consumption.

We investigated the packet collection ratio, that is, the number of packets received at the sink node divided by the total number of generated packets. We also investigated the energy consumption of the node with the heaviest load, which is determined by the maximum energy consumption, as well as the average energy consumption for all nodes when the packet generation rate (the number of data packets generated at each node per 1.0 s) is changed. Here, we use the term ‘performance’ to indicate the packet collection ratio, the maximum energy consumption, and the average energy consumption.

### 5.1. Basic Performance

The performance of all methods is examined for the topology shown in Figure 11. In order to investigate the basic performance, the intermittent interval is set to a constant value which is the same for all nodes. Although shorter intermittent intervals are important for improving the performance in IRDT, extremely short intervals cause frequent transmission of IDs, which appears to interfere with other communication. Therefore, we examine the basic performance in the case where the intermittent interval is set to 0.1 and 1.0 s. We clarify the performance characteristics of IRDT by comparing them with those of RI-MAC and X-MAC. In IRDT, each node transmits an ID packet and waits for an SREQ packet. The time for ID transmission is 1.92 ms, *T_ws_* is set to 2 ms, and in X-MAC each node periodically waits for 4 ms for a short preamble. In addition, X-MAC and RI-MAC use minimum hop routing, where sender nodes select one receiver node out of the neighboring nodes with minimum hop count from the sink node.

#### Packet collection ratio

**Figure 12.**
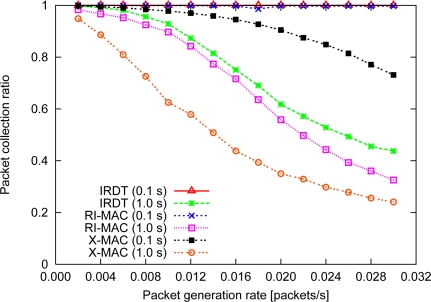
Packet collection ratio.

The collection ratio is shown in Figure 12. In case the intermittent interval is set to 0.1 s, highly frequent ID transmissions interfere with the communication of other nodes in IRDT and RI-MAC. However, the collection ratio is comparatively high (always over 98%) since *T_d_* is much longer than 0.1 s, which increases the chance for retransmission. In contrast, at an intermittent interval of 1.0 s, IRDT can attain a collection ratio of almost 100% when the packet generation rate is low, although the collection ratio decreases to less than 45% at relatively high packet generation rates. This result can be explained with SREQ collisions and the repeated SREQ collisions mentioned in Section 4.2. As the intermittent interval becomes longer, these collisions increase further, and the collection ratio for high packet generation rates at 1.0 s results in lower values of the collection ratio. Also, in RI-MAC, data packets collide with each other, and the packet collection ratio decreases as the packet generation rate increases. In this case, owing to the detour routing, IRDT can attain a higher packet collection ratio in comparison to RI-MAC.

In X-MAC, the collection ratio is lower than that in IRDT since the sender nodes transmit preamble packets without considering their receivers. If a sender node cannot obtain an early ACK, it transmits preambles throughout *T_d_*, which interferes with other communication. Thus, it can be said that X-MAC is clearly disadvantageous for retransmission in the MAC layer. However, unlike IRDT, in X-MAC a short intermittent interval does not interfere with other communication since each node periodically inspects the condition of the channel. Therefore, X-MAC can reduce the length of the intermittent intervals, and as a result it can achieve a higher collection ratio.

#### Energy consumption

**Figure 13.**
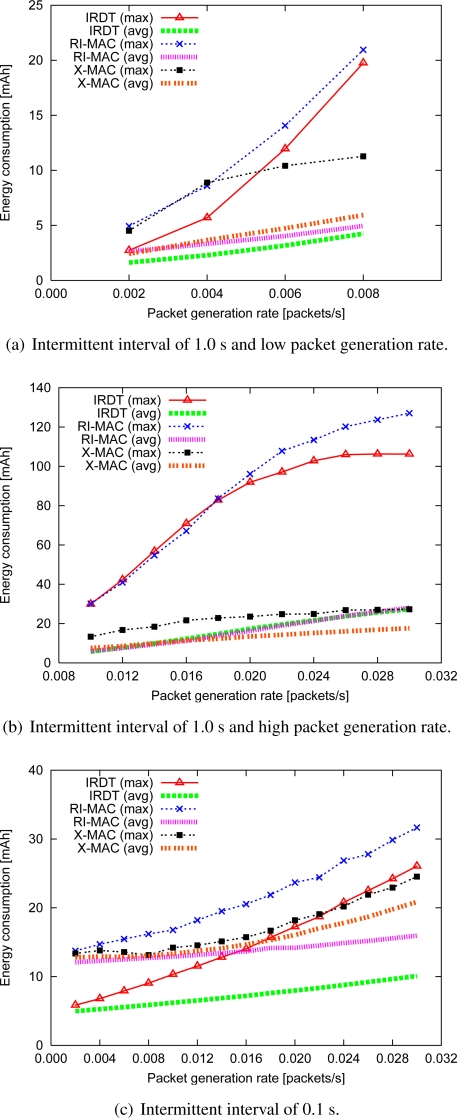
Energy consumption.

We examine the average energy consumption and the maximum energy consumption for all nodes (Figure 13).

In a comparison between IRDT and X-MAC at a low packet generation rate, when the intermittent interval is 1.0 s, the average energy consumption for IRDT is 33% lower than that of X-MAC since in IRDT there can be more than one receiver, as shown in Figure 13(a). In intermittent operations, more energy is consumed when sender nodes wait for the receiver, and using multiple receivers can reduce this waiting time. In comparing IRDT and RI-MAC, it is found that the energy efficiency of IRDT is higher due to the use of SREQ packets. Since the data packet size is larger than the SREQ packet size, when a receiver obtains a data packet and detects bit errors in RI-MAC after an ID transmission, the wasted energy is greater than that of SREQ collisions in IRDT. Also, in both IRDT and RI-MAC, the neighboring nodes of the sink node consume large amounts of energy since SREQ (or data) collisions occur more frequently at the sink node, which prolongs the idle time for listening for senders (Figure 13(b)). Thus, the energy consumption of the neighboring nodes of the sink node (IRDT (max)) grows rapidly in accordance with the increase of the packet generation rate when the intermittent interval is 1.0 s. Similarly, the energy consumption increases at nodes whose receivers experience frequent collisions of SREQ packets. In X-MAC, procedures for collision avoidance are not used, with the exception of CSMA/CA. Therefore, a short intermittent interval is necessary in order to achieve a higher collection ratio, although this prolongs the total idle listening time.

When the intermittent interval is 0.1 s, the maximum energy consumption in the case of IRDT does not grow considerably due to the smaller number of SREQ collisions (Figure 13(c)), and this is the same in the case of RI-MAC. The consumption of energy for both RI-MAC and X-MAC is higher than for IRDT. In RI-MAC, nodes wait for a data packet after sending an ID packet during *T_wd_*. This entails higher energy consumption than for IRDT, which uses *T_ws_*. In addition, energy is consumed by overhearing a short preamble or a data packet in X-MAC. Also, in X-MAC, each node attempts to transmit a short preamble packet without considering the state of the receivers, which results in data retransmissions and consequently increases the network-wide energy consumption.

### 5.2. Effects on Collision Avoidance for Control Packets

#### Reactive and proactive setting of the intermittent interval

At this stage, we introduce a method for SREQ collision avoidance (as described in Section 4.1 and 4.2) to IRDT and show the strong effects of this method. For the evaluation of this method for SREQ collision avoidance, we assume that exchanges of routing tables are not considered and all nodes have correct routing tables. The reactive setting of the intermittent interval is shown in Figure 7, and its parameters are shown in Table 2. *T_max_* is set by assuming continuous operation of about several years, and a short *T_min_* is set in order to reduce SREQ collisions. After the interval becomes *T_min_*, it is increased in steps of *T_i_* at every transmission of an ID packet. On the other hand, the proactive method uses T*. Here, as previously discussed in Section 4.2, each node can obtain the approximate value of T* by calculating the minimum value of Equation (7).

**Figure 14.**
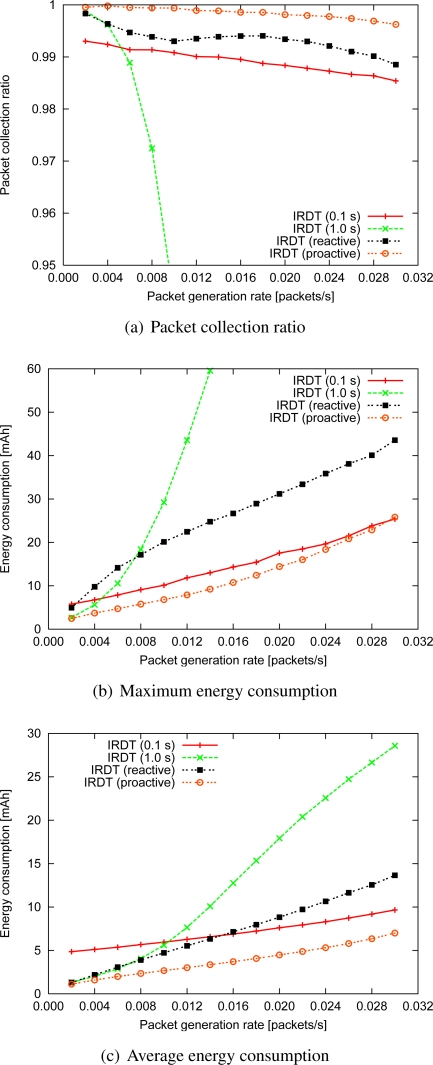
Improved performance of IRDT.

By avoiding control packet collisions, a higher collection ratio and lower energy consumption are achieved. In particular, the collection ratio in the proactive method is over 99.5% even when the packet generation rate is 0.030 (Figure 14(a)). This result indicates that IRDT can perform efficiently even at comparatively high packet generation rates.

Regarding the maximum energy consumption, its increase can be suppressed by using the proactive method with an interval of T*, as shown in Figure 14(b), due to the prevention of control packet collisions. Although the reactive method can also reduce energy consumption, except in the case of a packet generation rate of 0.002, it consumes larger amounts of energy than the original IRDT with the 0.1 s interval since the reactive mechanism attempts to avoid collisions after at least one collision has occurred. If SREQ collisions tend to occur in the neighboring nodes of the sink, for example, if there is a large number of such nodes, the improved IRDT is more effective even than the original at nodes adjacent to the sink. Additionally, preventing recurring SREQ collisions and shortening the ID waiting time can decrease energy consumption.

An intermittent interval of T* results in a 50% reduction of the maximum energy consumption as compared with the reactive setting of the intermittent interval at a packet generation rate of 0.002. Although a 40% reduction in energy consumption is also achieved at a packet generation rate of 0.030, with the proactive method the consumption of energy is as high as with the original IRDT with an interval of 0.1 s.

Since the shorter intermittent interval derived from T* yields greater chances of receiving data packets, this leads to implosion of the traffic. Therefore, a load balancing mechanism is necessary in order to reduce the maximum energy consumption, and this issue will be investigated in our future research.

Regarding the average energy consumption, when packets are generated infrequently, both the proactive and the reactive method suppress the increase in energy consumption, while at intermittent intervals of higher frequency, the reactive method consumes more energy than the original IRDT with an interval of 0.1 s. In addition, a reduction of 15% and 48% in average energy consumption is attained when the packet generation rate is 0.002 and 0.030, respectively.

#### Data aggregation

**Figure 15.**
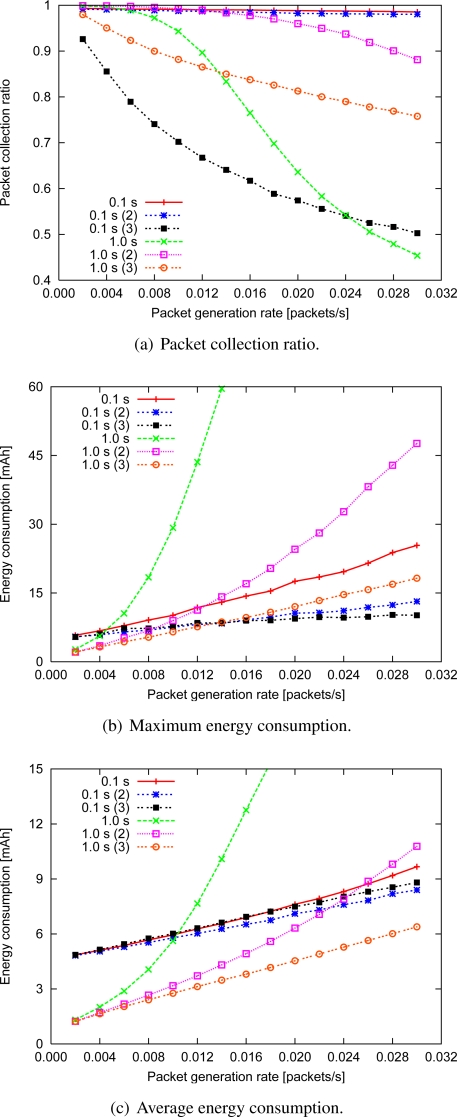
Performance with data aggregation.

The performance of IRDT with the data aggregation function is shown in Figure 15, where the number in the label denotes how many data packets can be included in a single aggregated data packet. Immediately after the reception or generation of data, each node waits for 5.0 s for aggregation without forwarding. When the intermittent interval is 1.0 s, the packet collection ratio increases with data aggregation (up to two data packets), after which it deteriorates with aggregation of three or more data packets. At an intermittent interval of 0.1 s, data aggregation always decreases the collection ratio since large data packets are likely to collide with ID packets. Moreover, the loss of aggregated data packets greatly decreases the collection ratio. In summary, our conclusion on the collection ratio is that aggregation of up to two data packets is effective in terms of avoidance of SREQ collisions, while aggregation of three or more packets is disadvantageous.

The maximum and the average energy consumption in all cases other than ‘0.1 s (3)’ decreases as the number of aggregated data packets increases [Figure 15(b) and 15(c)]. However, when the packet generation rate is low, data aggregation seldom occurs during the waiting time of 5.0 s, and the energy efficiency does not increase considerably.

Note that the increase in average energy consumption for the ‘0.1 s (3)’ case indicates that the increase in retransmissions due to ID collisions increases the number of data retransmissions everywhere in the network. For aggregation of up to three data packets when the packet generation rate is 0.030, a reduction in the maximum energy consumption of 83% and a reduction in the average energy consumption of 77% can be attained at an intermittent interval of 1.0 s. Moreover, the respective reduction of the maximum and the average energy consumption is 60% and 10% at an interval of 0.1 s. These improvements are achieved in particular by forwarding data to sideward nodes, which effectively suppresses SREQ collisions in nodes adjacent to the sink.

#### Combinations of intermittent interval setting and data aggregation

**Figure 16.**
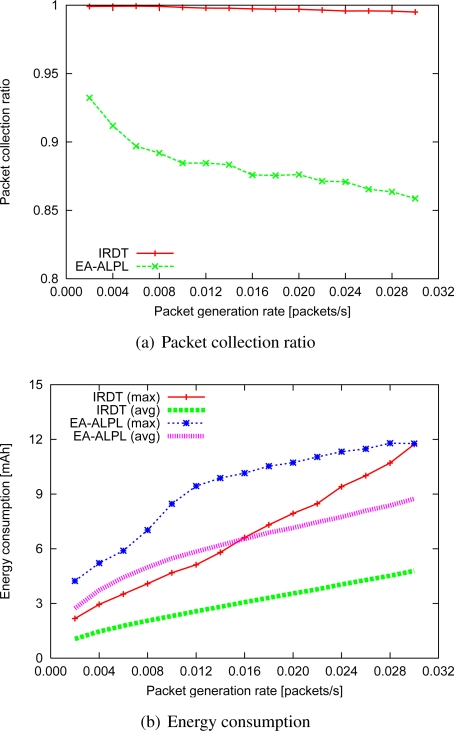
Performance using data aggregation and T*.

We compare the performance of IRDT with both the proactive collision avoidance method and data aggregation with that of EA-ALPL [9] as described in Figure 16, where data aggregation is limited to two data packets to prevent the packet collection ratio from decreasing. To conduct a fair comparison, EA-ALPL also uses data aggregation and an appropriate intermittent interval which minimizes the energy consumption (although it does not minimize packet collisions). However, due to the MAC layer protocol (B-MAC) of EA-ALPL, the intermittent interval is limited to 8 values (10, 20, 50, 100, 200, 400, 800, 1600 ms) [4]. Therefore, out of these eight values, EA-ALPL selects the value that is closest to the appropriate interval.

The results show that IRDT attains a higher collection ratio than EA-ALPL. In addition, IRDT has lower maximum and average energy consumption at all times, as seen in Figure 16(b). Specifically, the maximum and the average energy consumption at a packet generation rate of 0.002 can be reduced by 61% and 38%, respectively, although those at a packet generation rate of 0.030 can be reduced by only 0.1% and 45%, respectively. Moreover, a 90% reduction of the maximum energy consumption and an 84% reduction of the average energy consumption is achieved as compared with the original IRDT at an intermittent interval of 1.0 s. It is important to lower the maximum energy consumption for long-term operation of the network, and in this regard the avoidance of control packet collisions is highly efficient.

## 6. Conclusions

In this paper, we studied the basic performance characteristics of the receiver-driven asynchronous system IRDT. We also investigated the relation between control packet collisions and the intermittent interval and examined the efficacy of two simple settings of the intermittent interval and data aggregation in a comparison between IRDT, RI-MAC, and X-MAC, which is a sender-driven asynchronous system, by constructing a computer simulation. As a result, a reduction of 33% in the average energy consumption was achieved with IRDT as compared with RI-MAC and X-MAC. Furthermore, as compared with the original IRDT, the maximum energy consumption was reduced by 90%, and the average energy consumption was reduced by 84%.

At the present stage, our concern is that the comparison with other sender-driven methods, the load balancing for saving energy, and the handling time introduce changes into the network topology as a result of conditions imposed on the wireless channel. We believe that the maximum energy consumption, which is closely related to the network lifetime, can be reduced further by employing load balancing. In terms of changes in the network topology, we expect that robust networks can be built with IRDT due to its promising capabilities for constructing mesh networks, although we do not consider any interchange and update of topological information. These issues will be addressed in our future work.

## Figures and Tables

**Table 1.** Parameter settings.

Parameter	Value
Simulation time	6 h
*T_d_*	5 s
*T_si_*	300 s
*TTL*	*H* + 3
*T_ws_*	2 ms
*T_wd_*	10 ms
Contention window size (*W_min_*)	3
Contention window size (*W_max_*)	5
Current consumption (TX)	20 mA
Current consumption (RX)	25 mA
Current consumption (Sleep)	0 mA
Packet size (ID, SREQ)	24 bytes
Packet size (DATA)	128 bytes
Packet size (RACK, DACK)	22 bytes
Transmission rate	100 kbps

**Table 2.** Parameter settings.

Parameter	Value
*T_max_*	1.5 s
*T_min_*	0.1 s
*T_i_*	10 ms
*P_f_*	50%
